# Retrospective validation of G-ROP, CO-ROP, Alex-ROP, and ROPscore predictive algorithms in two Chinese medical centers

**DOI:** 10.3389/fped.2023.1079290

**Published:** 2023-02-22

**Authors:** Yang Lu, Zhe Lv, Jiner Cen, Jiwei Tao, Yun Zhang, Yifan Zhang, Jianbo Mao, Yiqi Chen, Mingyuan Wu, Shujun Chen, Lijun Shen

**Affiliations:** ^1^ National Clinical Research Center for Ocular Diseases, Eye Hospital, Wenzhou Medical University, Wenzhou, China; ^2^ Department of Ophthalmology, Lishui People’s Hospital, Lishui, China; ^3^ Department of Ophthalmology, Jiaxing Second People's Hospital, Jiaxing, China; ^4^ Department of Ophthalmology, Zhejiang Provincial People's Hospital, Hangzhou, China; ^5^ Department of Neonatology and Pediatrics, Women’s Hospital School of Medicine Zhejiang University, Hangzhou, China; ^6^ Department of Neonatology and Pediatrics, Yiwu Maternity and Children Hospital, Yiwu, China

**Keywords:** retinopathy of prematurity, G-ROP, CO-ROP, prediction model, algorithm, infants, validation

## Abstract

**Purpose:**

To evaluate the sensitivity and specificity of four predictive algorithms (G-ROP, CO-ROP, Alex-ROP, and ROPscore) for retinopathy of prematurity and compare their performances in the Chinese population.

**Methods:**

A retrospective study was conducted at two medical centers in China of infants born at Women's Hospital School of Medicine Zhejiang University and Yiwu Maternal and Child Health Hospital. A total of 1,634 infants who met the criteria and who were GA < 32 weeks or BW < 2,000 g according to Chinese guidelines for ROP screening were included. The ROP group was further grouped into severe ROP and mild ROP. The sensitivity and specificity of G-ROP, two simplified G-ROPs, CO-ROP, Alex-ROP, and ROPscore were analyzed.

**Results:**

Severe ROP and any ROP were identified in 25 and 399 of 1,634 infants, respectively. According to the criteria of different models, 844, 1,122, 1,122, and 587 infants were eligible in the G-ROP, CO-ROP, Alex-ROP, and ROPscore, respectively. G-ROP had 96.0% sensitivity and 35.0% specificity for severe ROP. For two simplified G-ROPs (180 g and 200 g models), similar sensitivity was showed with original G-ROP and they had specificity of 21.8% and 14.0%, respectively. The sensitivity and specificity of Co-ROP were 96% and 64.3% for severe ROP, while Alex-ROP only had sensitivity of 56.0% and specificity of 61.4% for severe ROP. ROPscore had a sensitivity of 91.3% and a specificity of 62.4% for severe ROP. In 546 infants who met all 4 models' inclusion criteria and included 23 infants with severe ROP, the validation outcomes showed the sensitivity of G-ROP, ROPscore, CO-ROP, and Alex-ROP for severe ROP was 95.6%, 91.3%, 100%, and 56.0%, and their specificity was 38.0%, 60.8%, 39.9%, and 52.9%, respectively.

**Conclusion:**

G-ROP, ROPscore, and CO-ROP had high sensitivity for severe ROP in the Chinese population, but both the sensitivity and specificity of Alex-ROP were low. CO-ROP (not high-grade CO-ROP) provided the best performance for severe ROP in a fair comparison. For further application, ROP screening models need to be adjusted by local populations.

## Background

Retinopathy of prematurity (ROP) is a leading disease that causes blindness in premature infants around the world. However, it is preventable in the early period by laser retinal photocoagulation or intravitreal injection of antivascular endothelial growth factor (1). Therefore, it is important to screen ROP in those infants with a high risk of ROP. Extensive research has shown that gestational age (GA) and birth weight (BW) are the two most important factors of ROP (2). Screening guidelines based on GA and BW were employed in many countries, such as the criteria that GA < 32 weeks or BW < 2,000 g in China and GA < 30 weeks or BW < 1,500 g in America (3). However, although those screening guidelines performed high sensitivity, the majority of infants received unnecessary examination, which needed a lot of human and material resources. Recent evidence suggested that postnatal weight gain could be a contributing factor to the development of ROP (4). Thus, many ROP prediction models based on weight gain were developed to improve the efficiency of ROP screening (5–8).

Postnatal Growth and Retinopathy of Prematurity (G-ROP) Criteria was developed by Binenbaum et al. with a large sample size, which involved weight gain in 3 discrete periods (5). Cao et al. calculated net weight gain between birth and 1 month of age to develop Colorado–retinopathy of prematurity model (CO-ROP) (6). Similarly, the net weight gain ratio (NWGR) at 28 days after birth was applied in the Alexandria retinopathy of prematurity model (Alex-ROP), which was implemented in a developing country (7). However, the three models above have not yet been validated in the population of the Chinese mainland. ROPscore proposed by Eckert et al. is a scoring calculation including weight gain at 6 weeks after birth, blood transfusions, and use of mechanical ventilation except for GA and BW (8). ROPScore has been applied in many developing countries, showing varying feasibility (9, 10).

Due to the differences in the neonatal care systems among different countries and the influence of race in the development of ROP, ROP prediction models might perform differently. The specific objective of this study was to validate G-ROP, Co-ROP, Alex-ROP, and ROPscore to explore their feasibility in the Chinese population.

## Methods

### Patients

A bicentric retrospective study of infants born at Women's Hospital School of Medicine Zhejiang University in two periods (January 2016 to April 2019, August 2019 to June 2020) and Yiwu Maternal and Child Health Hospital from November 2016 to May 2018 was conducted and approved by the local ethics committees. Premature infants who met the criteria that were GA < 32 weeks or BW < 2,000 g for ROP were screened. Infants with unknown ROP outcome and other ocular abnormality were excluded.

### ROP screening

RetCamIII was used to screen the fundus after pupil dilatation by a professionally trained physician. The screening time, classification, and treatment of ROP were carried out according to China's screening guidelines (11). Mild ROP was defined as ROP at stage 1 or stage 2 in zone II or III without plus disease and ROP at stage 1 in zone III with plus disease. Severe ROP was defined as Type 1 ROP, Type 2 ROP, and threshold ROP, according to CO-ROP, Alex-ROP, and local guidelines. For type 1 ROP, treatment should be considered as early as possible. The criteria for treatment in type 2 ROP were listed as follows (12, 13): zone 2 stage 3 with neovascularization or ridge with anteroposterior traction for progression to stage 4 disease; Zone 2 stage 3 with pre-plus disease; persistent zone 2 stage 3 ROP that showed no evidence of regression for 6 weeks; zone 2 stage 3 and zone 1 stage 2 disease with type 1 ROP in the fellow eye. Record the worst screening result of an infant in all of his examinations as his ROP outcome. For all infants who were screened, the researchers told their parents about the risks and precautions of screening and gave written informed consent.

### Data collection

Demographic and medical data including GA, BW, weight gain, hydrocephalus, mechanical ventilation, blood transfusions, and ROP outcome at every examination were collected. Infants born at 28 weeks and 6 days would be considered as 28 + 6/7 weeks, and so on. Diagnosis of hydrocephalus was dependent on B-ultrasound or MRI. Weight measurement was conducted every 2 or 3 days in NICU and if no weight was recorded at a specified point of time, weight would be calculated by previous and next weight under the assumption that weight changed linearly from previous to next day where weight was measured.

### Models screening

G-ROP requires 6 criteria as followed: GA < 28 weeks; BW < 1,051 g; weight gain (WG) between day 10 and 19 after birth is less than 120 g; WG between day 20 and 29 after birth is less than 180 g; WG between day 30 and 39 after birth is less than 170 g; hydrocephalus. For all of the infants screened, 6 criteria would be checked one by one and once the infant met one of the criteria, examination should be considered. 2 modified G-ROP screening criteria, which used 180 g or 200 g across the three 10-day periods would also be validated.

In Co-ROP screening, infants meeting three criteria at the same time would receive examinations: GA ≤ 30 weeks, BW ≤ 1,500 g, and net weight gain (NWG) ≤ 650 g between birth and 1 month of age. NWG ≤ 400 g would be considered as a high risk of high-grade ROP (Hg CO-ROP) (6). In the Alex-ROP model, infants with GA ≤ 33 weeks or BW ≤ 1,500 g and NWGR < 0.3 at postnatal day 28 were suggested to take the exam, and High-grade Alex-ROP (Hg Alex-ROP) model suggests detecting worse grade ROP (both type 1 and type 2) for infants with NWGR < 0.15 at postnatal day 28 (7). ROPscore is a linear regression calculation containing the following variables: BW(g), GA(w), WG(g) at 6 weeks after birth, mechanical ventilation, and blood transfusions with coefficients of −0.004, −0.263, −1.258, +1.920 and +1.980, respectively. Cut-off values for any stage of ROP and severe ROP were 11.0 and 14.5 in the original study (8). Adjusted cut-off values would be calculated according to the population in this study.

**Figure 1:**
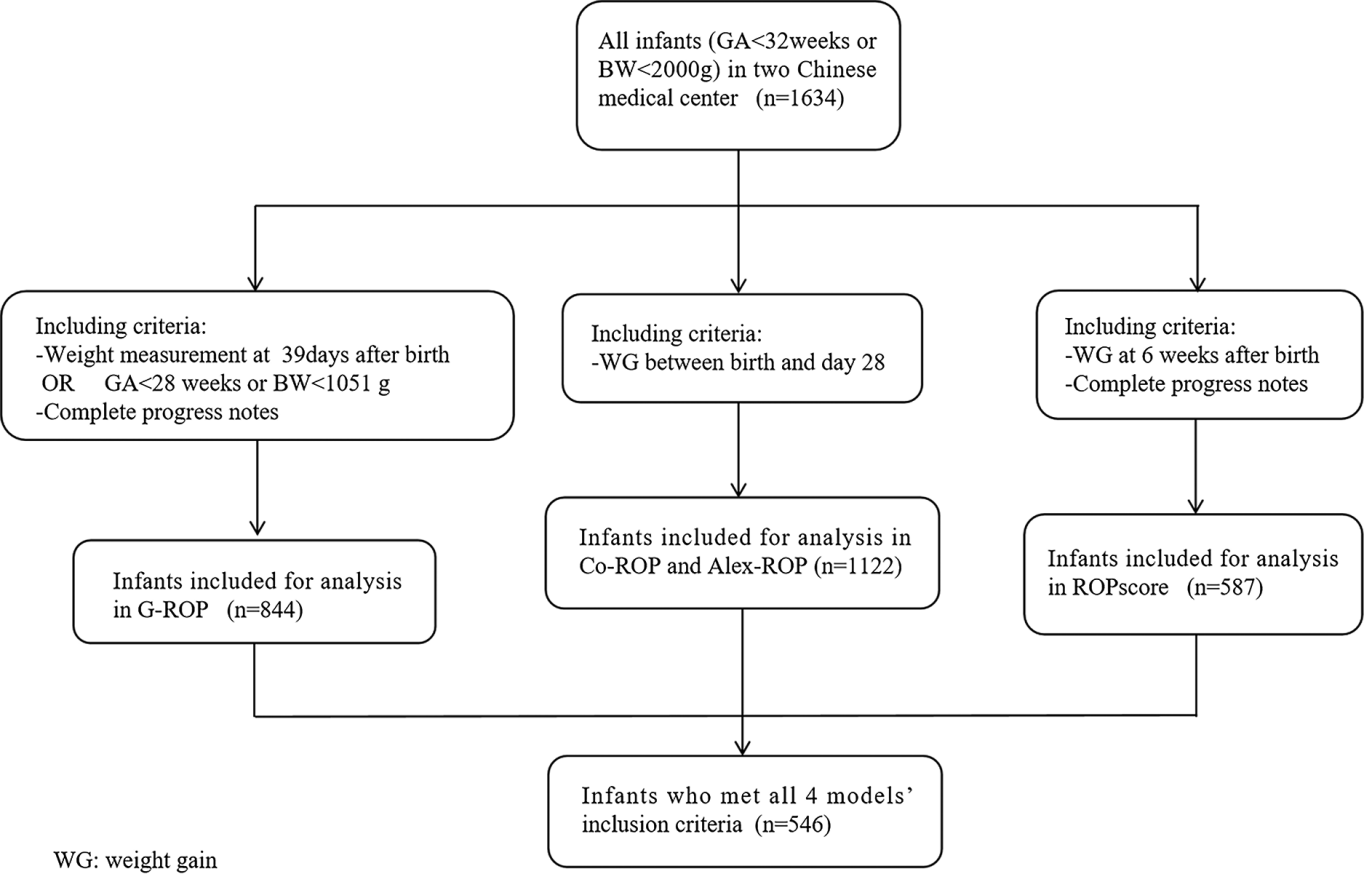
Flow diagram showing the including criteria for the infants in different models.

For each prediction model, infants who lacked the necessary information were excluded from the population. To have a fair comparison, the population who met al.l four models’ inclusion criteria was also validated throughout the 4 models. Figure 1 showed the flow diagram of including criteria in different models.

### Statistical analysis

Sensitivity and specificity for any ROP and severe ROP in each prediction model were calculated. Wilson score method was utilized to get the 95% confidence intervals (CIs) of sensitivity and specificity. To get better predictive performance in the Chinese population, receiver operating characteristic (ROC) curves of the ROPscore were conducted and Area Under Curve (AUC) was recalculated. For ROPscore, we set the new cut-off values for any ROP and severe ROP. For severe ROP, a new cut-off value was set on the premise of maintaining the 100% sensitivity of the model and reducing the number of screening infants as much as possible. For any ROP, according to the sensitivity and specificity of other ROP models, the range of above 90% sensitivity and above 30% specificity was established, and in this range, the new cut-off value was set when sensitivity plus specificity reached its maximum. SPSS version 23.0 and R version 4.1.0 were used for statistical analysis.

## Results

### Characteristics of population

A total of 1,634 infants were screened for ROP in two hospitals during 3 periods. According to the criteria of different models, 844, 1,122, 1,122, and 587 infants were eligible in the G-ROP, CO-ROP, Alex-ROP, and ROPscore, respectively. For G-ROP, CO-ROP and Alex-ROP, and ROPscore, the average GA and BW of excluded infants were 32.1 weeks and 1,632.5 g, 32.1 weeks and 1,618.2 g, and 31.8 weeks and 1,588.2 g. There were 546 infants who met all 4 models' inclusion criteria. Some of the main characteristics of the diverse population in different models were summarized in Table 1.

**Table 1:** Characteristics of infants included in G-ROP, CO-ROP, Alex-ROP and ROPscore.

	All infants	Severe ROP	Any ROP	No ROP
**G-ROP**
*n*	844	25	315	529
GA				
Mean (SD)	29.9 ± 2.1	27.2 ± 1.2	28.8 ± 1.9	30.6 ± 1.9
Median (range)	30.0 (24.2, 39.5)	27.2 (25.0, 30.1)	28.7 (24.2, 39.5)	30.71 (25.0, 37.5)
BW				
Mean (SD)	1,327.4 ± 307.6	955.0 ± 233.7	1,167.4 ± 260.2	1,422.6 ± 294.1
Median (range)	1,320 (600, 2,880)	910 (670, 1,820)	1,150 (600, 2,880)	1,410 (650, 2,330)
**co-ROP&Alex-rop**
*n*	1,122	25	325	797
GA				
Mean (SD)	30.5 ± 1.9	27.2 ± 1.2	29.2 ± 1.9	31.0 ± 1.7
Median (range)	30.7 (25.0, 39.5)	27.2 (25.0, 30.1)	29.1 (25.0, 39.5)	31.0 (25.0, 36.7)
BW				
Mean (SD)	1,406.1 ± 299.9	955.0 ± 233.7	1,214.0 ± 263.8	1,484.4 ± 277.8
Median (range)	1,390 (600, 2,330)	910 (670, 1,820)	1,190 (600, 2,050)	1,500 (650, 2,330)
**Ropscore**
*n*	587	23	250	337
GA				
Mean (SD)	29.5 ± 1.6	27.1 ± 1.1	28.8 ± 1.6	30.0 ± 1.4
Median (range)	29.7 (25.0, 34.0)	27.2 (25.0, 29.8)	28.7 (25.0, 33.0)	30.0 (25.0, 34.0)
BW				
Mean (SD)	1,272.2 ± 271.7	922.3 ± 154.4	1,155.9 ± 228.7	1,358.5 ± 269.2
Median (range)	1,260 (600, 2,130)	910 (670, 1,250)	1,145 (600, 1,900)	1,360 (600, 1,900)
**All models**
*n*	546	23	238	308
GA				
Mean (SD)	29.4 ± 1.7	27.1 ± 1.1	28.7 ± 1.7	29.9 ± 1.5
Median (range)	29.4 (25.0, 34.0)	27.2 (25.0, 29.8)	28.4 (25.0, 33.0)	30.0 (25.0, 34.0)
BW				
Mean (SD)	1,258.6 ± 270.2	922.3 ± 154.4	1,139.5 ± 218.8	1,350.7 ± 270.4
Median (range)	1,240 (600, 2,130)	910.0 (670, 1,250)	1,130 (600, 1,690)	1,360 (750, 2,130)

BW, birth weight; GA, gestational age; ROP, retinopathy of prematurity; SD, standard deviation; WG, weight gain.

### Validation outcome

**Figure 2:**
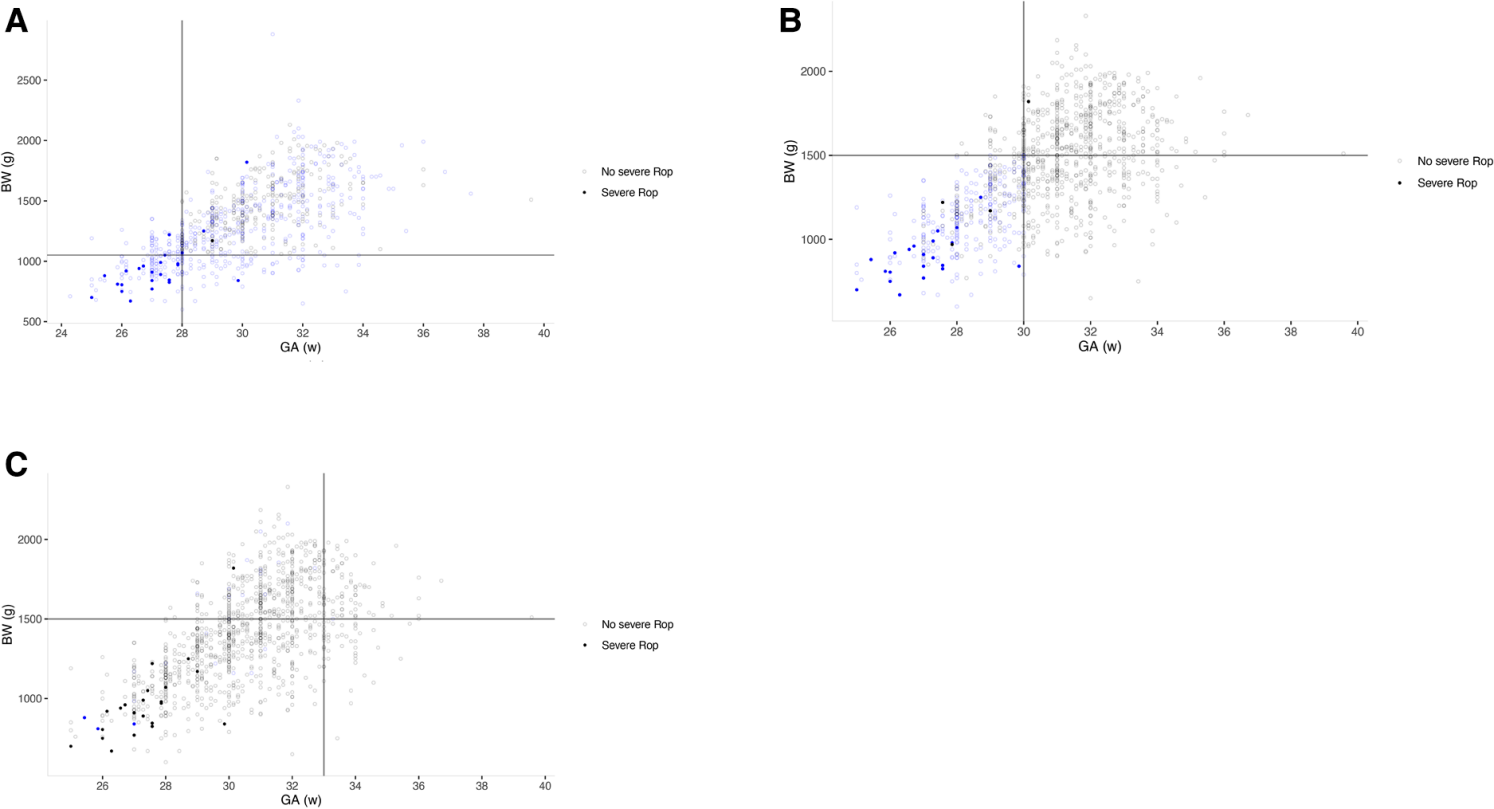
Three scatter plots based on gestational age (GA) and birth weight (BW) of infants in three populations. Empty circle means infants with severe ROP, and filled circle means infants without severe ROP. Circles color-coded by blue were infants included by each model. The lines define the thresholds for BW and GA in different models. (**A**) G-ROP for severe ROP; (**B**) high-grade Co-ROP for severe ROP; (**C**) High grade Alex-ROP for severe ROP.

The G-ROP reduced the number of infants screened by 34.2% compared to current China's screening guidelines. The model had a sensitivity of 96.0% (95%CI: 80.4%–99.2%) for severe ROP. As shown in Figure 2A in which infants were classified by distribution of GA and BW in G-ROP distinguished 22 severe ROP, and 2 severe ROP were predicted by low postnatal weight gain, while 1 severe ROP was missed. The G-ROP 180 g model and G-ROP 200 g model both had a sensitivity of 96.0% and their specificity was 21.8% and 14.0%, respectively.

**Figure 3:**
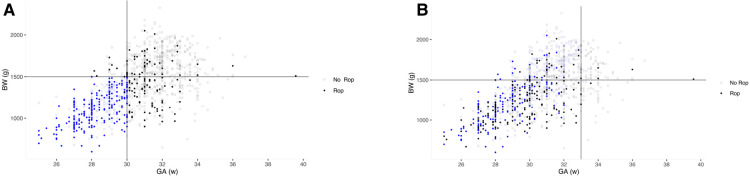
Two scatter plots based on gestational age (GA) and birth weight (BW) of infants in two populations. Empty circle means infants with any ROP, and filled circle means infants without any ROP. Circles color-coded by blue were infants included by each model. The lines define the thresholds for BW and GA in different models. (**A**) Co-ROP for any ROP; (**B**) Alex-ROP for any ROP.

The number of infants screened decreased from 1,122 to 365 in CO-ROP, reducing 63.0% of infants. The sensitivity was 96.0% (95%CI: 80.4%–99.2%) and 65.5% (95%CI: 60.2%–70.4%) for severe ROP and any ROP, and the specificity for no severe ROP and no any ROP was 64.3% (95%CI: 61.4%–67.1%) and 74.6% (95%CI: 71.5%–77.5%). There was 1 severe ROP missed by CO-ROP. However, Alex-ROP only had sensitivity of 56.0% (95%CI: 37.0%–73.3%) and 45.3% (95%CI: 40.0%–50.8%) for severe ROP and any ROP, and high-grade Alex-ROP had a lower sensitivity of 12.0% (95%CI: 4.1%–29.9%) by screening only 25 of 1,122 infants (2.22%). As shown in Figures 3A,B, the sensitivity of Co-ROP for any ROP was high in the infants with GA ≤ 30 weeks and BW ≤ 1,500 g, while many infants with any ROP were missed according to the criteria of WGR in Alex-ROP. In fact, 215 ROP developed in 422 infants with GA ≤ 30 weeks and BW ≤ 1,500 g, and 415 infants required screening with 213 ROP detected when criteria of WG less than 650 g in 28 days was applied, missing 2 ROP by reducing 7 infants in CO-ROP. Table 2 shows the performance of G-ROP, CO-ROP, and Alex-ROP.

**Table 2:** Prediction of severe ROP and any ROP using G-ROP, CO-ROP and Alex-ROP.

Model	G-ROP	Co-ROP	hgCO-ROP	Alex-ROP	High grade Alex-ROP
*N*	844	1,122	1,122	1,122	1,122
**Severe ROP**					
Sensitivity (95%CI)	96.0% (80.4%, 99.2%)	96.0% (80.4%, 99.2%)	84.0% (65.3%, 93.5%)	56.0% (37.0%, 73.3%)	12.0% (4.1%, 29.9%)
Specificity (95%CI)	35.0% (31.8%, 38.3%)	64.3% (61.4%, 67.1%)	77.3% (74.7%, 79.6%)	61.4% (58.5%, 64.2%)	97.9% (96.9%, 98.6%)
**Any ROP**					
Sensitivity (95%CI)	−	65.5% (60.2%, 70.4%)	−	45.3% (40.0%, 50.8%)	−
Specificity (95%CI)	−	74.6% (71.5%, 77.5%)	−	63.6% (60.2%, 66.9%)	−
*N*	546	546	546	546	546
**Severe ROP**	** **	** **	** **	** **	** **
Sensitivity (95%CI)	95.6% (79.0%, 99.2%)	100.0% (85.6%, 100%)	86.9% (73.2%, 97.5%)	56.0% (36.8%, 74.3%)	8.6% (2.4%, 26.7%)
Specificity (95%CI)	38.0% (33.9%, 42.2%)	39.9% (35.8%, 44.2%)	60.2% (55.9%, 64.3%)	52.9% (48.6%, 57.2%)	98.2% (95.3%, 99.0%)
**Any ROP**					
Sensitivity (95%CI)	−	78.1% (72.4%, 82.9%)	−	44.1% (37.9%, 50.4%)	−
Specificity (95%CI)	−	59.0% (50.2%, 61.7%)	−	50.0% (44.0%, 55.5%)	−

hgCO-ROP, high-grade CO-ROP.

Although ROPscore predicted 97.2% any ROP (95%CI: 94.3%–98.6%), it required 92.5% of the infants to be screened with very low specificity. For severe ROP, 233 of 587 infants would be screened with a sensitivity of 91.3% (95%CI: 73.0%–97.5%). According to the population in our study, the AUC for severe ROP and any ROP were 0.90 and 0.70, respectively. The new optimal cut-off values to predict any ROP and severe ROP were 12.39 (90.8% sensitivity and 32.3% specificity) and 13.68 (100% sensitivity and 48.4% specificity) in our study. Table 3 showed performance of ROPscore and adjusted ROPscore. Table 4 shows the characteristics and other possible risk factors of missed infants by G-ROP, CO-ROP, and ROPsocre.

**Table 3:** Prediction of severe ROP and any ROP using ROPscore and adjusted ROPscore.

	Severe ROP		Any ROP	
	Sensitivity (95%CI)	Specificity (95%CI)	Sensitivity (95%CI)	Specificity (95%CI)
*N* = 587				
ROPscore (cutoff = 11)	100%(85.6%, 100%)	7%(5.0%, 10.3%)	97.2%(94.3%, 98.6%)	10.9%(8.0%, 14.7%)
ROPscore (cutoff = 14.5)	91.3% (73.0%, 97.5%)	62.4% (58.3%, 66.3%)	−	−
Adjusted ROPscore (cutoff = 12.39)	100% (85.6%, 100%)	23.4%(20.0%, 27.0%)	90.8%(87.6%, 94.3%)	32.3%(27.5%, 37.5%)
Adjusted ROPscore (cutoff = 13.68)	100%(85.6%, 100%)	48.4%(44.3%, 52.5%)	−	−
*N* = 546				
ROPscore (cutoff = 11)	100% (85.6%, 100%)	8.4% (6.3%, 11.1%)	98.3% (95.7%, 99.3%)	10.7% (7.7%, 14.6%)
ROPscore (cutoff = 14.5)	91.3% (73.2%, 97.5%)	60.8% (56.5%, 64.8%)	−	−
Adjusted ROPscore (cutoff = 12.39)	100% (85.6%, 100%)	20.8%(17.5%, 24.5%)	93.2%(89.3%, 95.8%)	30.1%(25.3%, 35.5%)
Adjusted ROPscore (cutoff = 13.68)	100% (85.6%, 100%)	46.0%(41.8%, 50.1%)	−	−

**Table 4:** Risk factors of the 4 infants with severe ROP undetected by different models.

	Undetected by G-ROP	Undetected by CO-ROP	Undetected by ROPscore	Undetected by ROPscore
GA (w)	29	30 + 1	28 + 5	28
BW (g)	1,170	1,820	1,250	1,070
Supplemental oxygen (days)	42	0	21	26
WG in 28 days	620	617	260	310
WG in 42 days	1,160	−	530	630
Use of mechanical ventilation	+	−	+	+
Blood transfusions	+	−	−	−
Neonatal feeding problem	+	−	−	−
Hydrocephalus	−	+	−	−
Test tube baby	−	−	+	+
One of twins	−	−	+	+
Congenital heart disease Necrotizing enterocolitis Bronchopulmonary dysplasia	−	−	−	+
	−	−	−	−
	−	−	−	−

In the row of GA (weeks), the numbers after “+” are in days. In the other rows, “+” means that the infant has the characteristic in the first column., and “−” means that the infant doesn’t have the characteristic in the first column.

In the validation of 546 infants who met all 4 models' inclusion criteria, for severe ROP, similar results were obtained in G-ROP and ROPscore, and CO-ROP had a sensitivity of 100%.

## Discussion

Our study validated 4 ROP prediction models based on postnatal weight gain in the Chinese population. Two models were developed in North America (G-ROP and CO-ROP). The other two models were established by developing countries (Brazil and Egypt). The different performance of the four models was revealed in our study.

Binenbaum et al. developed the G-ROP model in 2018 (5). This is a multicenter retrospective study based on a large sample size, including 7,483 premature infants from 29 hospitals in the United States and Canada from 2006 to 2012. The weight gain at three time periods was determined as a predictor except for GA and BW. Once the newborn meets one of the criteria, it can be included in the screening. Therefore, for premature infants with low BW, small GA, and low weight gain, fundus screening can be conducted as soon as possible. In the internal validation, G-ROP could reduce about 30.3% of the screening population (2,269/7,483), predicting 100% type 1 ROP (459/459) and treated ROP (524/524), and 98.7% type 2 ROP (466/472) (5).

In our study, G-ROP had a sensitivity of 96.0% for severe ROP and reduced the number of infants screened by 34.2%. The 3 severe ROP missed by GA and BW were captured by WG, which demonstrated that WG contributed to detecting severe ROP from relatively larger BW and older GA infants. More than 10 validation studies for G-ROP have been reported. In the population of developed countries (14, 15) (such as USA, Japan, and UK) where GA and BW were less due to strict screening criteria, higher sensitivity (almost 100%) was calculated because of less influence of other factors on ROP except for weight gain after birth. In the external validation of a North American population with 28.0 weeks of median GA and 1,072 g of median BW, G-ROP exhibited excellent performance with 100% sensitivity for type1 ROP and 98.6% sensitivity for type2 ROP by reducing the number of infants receiving examinations by 32.5% (14). For the Asian population, 537 infants with 29.1 weeks of median GA and 986 g of median BW were validated in Japan (15), resulting in 100% sensitivity for treated ROP (TR-ROP) by reducing infants screened by 24.5%. In a UK cohort with 29 weeks of median GA and 1,010 g of median BW, a sensitivity of 100% for type 1 ROP was obtained (16). On the other hand, broad screening criteria may lead to relatively greater GA and BW as in our study. In an Italian population with 30.4 weeks of median GA and 1,300 g of median BW, sensitivity for type 1 ROP, type 2 ROP, and any ROP was 100%, 93.7%, and 87.4% by G-ROP, respectively (17). Moreover, sensitivity of 91.2% and 88.3% was calculated for any ROP and TR-ROP in 242 infants with a mean GA of 29.5 weeks and a mean BW of 1,303.4 g in Turkey (18). A validation indicated a sensitivity of 100% for type 1 ROP in an Egyptian cohort with 31.5 weeks of median GA and 1,200 g of median BW (16). Our study has similar sensitivity and specificity to most studies, but still missed 1 severe ROP which was also missed in 2 modified G-ROP screening criteria in our study, as in other studies (14). In a validation of a large sample size of the North American population, 3 periods of 180 g G-ROP showed the same sensitivity for severe ROP with original G-ROP (14), which was similar to our study. However, 3 periods of 180 g G-ROP increased sensitivity for severe ROP from 96.6% to 100% of the population of Taiwan (19). In our study, the feeding problems of the missing one which led to the much nonphysiological weight gain were found, indicating that nonphysiological weight gain by some diseases may be the cause of omission.

Although G-ROP performed as well as other validation studies for severe ROP in our study, which is the main purpose of G-ROP, the 74.8% sensitivity for any ROP in our study is lower than the sensitivity in other validations. One of the reasons could be more infants with older GA, larger BW, and larger WG developed mild ROP in our population than in others.

Co-ROP and Alex-ROP both require GA, BW, and WG between birth and 28 days (net weight gain and net weight gain rate). Meanwhile, the two models both have high-grade models to detect severe ROP. However, the risk of ROP is only calculated at 28 days after birth, which means infants with a high risk of ROP may miss treatment time.

Although Co-ROP reduced infants screened by 63.0% with a sensitivity of 96% for severe ROP in our study, the performance in predicting any ROP was poor with a sensitivity of 65.5%. In the population of 546 infants who met all 4 models' inclusion criteria, CO-ROP provided the best performance for severe ROP with 100% sensitivity and 39.9% specificity. However, the performance of Alex-ROP was poorer with a sensitivity of 56.0% and 45.3% for severe and any ROP in our study. In the validation of Co-ROP in the G-ROP population, the sensitivity for severe ROP and any ROP was 96.9% and 92.8% in 6,351 infants, and the CO-ROP model would have eliminated ROP screening for 1,655 infants (26.1%) (20). Similarly, a multicenter study with a medium sample size in the US also resulted in good performance of Co-ROP for type 1 or type 2 ROP and any ROP (21).

Thus, Co-ROP showed great performance for severe ROP in several studies (including our study) even though it did not exhibit a sensitivity of 100% (20–22), while it was not suitable for predicting any ROP in our study due to many infants with older GA, larger BW, and larger WG who developed mild ROP in our study. In the secondary analysis of validation of CO-ROP in our study, criteria of WG≤ 650 g only reduced 7 screened infants and missed 2 ROP in infants with GA ≤ 30 weeks and BW ≤ 1,500 g, indicating that WG between birth and 28 days made few contributions to distinguishing infants with ROP.

Alex-ROP is a screening model established for developing countries where older infants with larger BW than developed countries (7), and our study was the first one to validate it. Unlike Co-ROP, NWGR at day 28 was used to replace NWG in the model. However, even though China is still a developing country, Alex-ROP performed poorly with a sensitivity of 56.0% for any ROP in the Chinese population, probably due to the relatively more developed neonatal care system in Chinese eastern coastal cities than in Egypt. As shown in Figure 3C, many infants with ROP were missed, indicating the criteria of weight gain ratio at 28 days after birth less than 0.3 was not applicable to many infants with ROP in our study.

ROPscore also requires weight gain at 6 weeks after birth, which is similar to CO-ROP and Alex-ROP (8). The ROPscore model has been validated in various regions (9, 10, 23–25) and the sensitivity of severe ROP varies between 73% to 100%. As a calculator to predict ROP, the cut-off value could be adjusted for different populations. New cut-off values for any ROP and severe ROP were 12.39 with sensitivity of 90.8% and specificity of 32.3% and 13.68 with sensitivity of 100% and specificity of 48.4% in our study. Recently, a study by Sun et al. validated ROPscore in a population of a Chinese city from 2009 to 2019 (25). For any stage of ROP, the AUC was 0.70 consistent with 0.70 in our study and the optimal cut-off point was 12.3 with sensitivity of 55.8% and specificity of 77.8%. However, for severe ROP, the AUC was 0.76 in their study, while 0.90 of AUC was calculated in our research, and the optimal cut-off value for severe ROP was 13.3 in Sun's research. ROPscore seemed to be more suitable for the population in our study to predict severe ROP, probably because of the small sample size of severe ROP in our study. Another possibility could be that level of neonatal care systems varied during the decade where infants were screened in Sun's research, and older infants with larger BW were more likely to develop severe ROP in the early period.

There were two severe ROP that didn't meet the inclusion criteria and were excluded in ROPscore. One severe ROP who had 27 weeks of GA, 840 g of BW, and 0.44 of WGR at 6 weeks lacked progress note. The result of score was 15.75 which was greater than 14.5, on the assumption that mechanical ventilation was used (as almost infants with low GA and BW like him was treated as mechanical ventilation) without blood transfusion. Another severe ROP had 30.14 weeks of GA and 1,820 g of BW without mechanical ventilation and blood transfusion, but was diagnosed with hydrocephalus left hospital when he was only born at 40 days. His weight gain was 728 g at 40 days after birth and corresponding WGR was 0.60. Thus, after 0.60 of WGR at 40days after birth replaced the WGR at 6 weeks after birth when calculating the equation of ROPscore, the result of the score was 8.87 which was much lower than 14.5, indicating that it was very likely that the infant with severe ROP would be missed by ROPscore when he has completed data. So potential sources of nonphysiological weight gain caused by hydrocephalus should be considered when models are used.

An apparent decrease was found in specificity for Co-ROP, hgCO-ROP, and Alex-ROP, when comparing with the specificity among their own cohorts. Infants of the population eligible for all four models had relatively smaller GA and BW compared with the cohorts of the three models above. So the GA and BW in the criteria of Co-ROP, hgCO-ROP(GA ≤ 30 weeks and BW ≤ 1,500 g), and Alex-ROP (GA < 33 weeks or BW < 1,500 g) were relatively extensive for the population eligible for all four models. Thus, a higher proportion of infants without ROP was in the common cohort for the three models, so the specificity for the three models was decreased compared with the specificity among their own cohorts.

Missed infants with severe ROP in models should get more attention, because the undetected severe ROP may cause irreversible visual impairment. The infant with severe ROP missed by G-ROP has been treated by mechanical ventilation and blood transfusions, which are two of the criteria in ROPsocre. Although he has 620 g and 1,160 g of WG in 28 and 42 days, his neonatal feeding problem leaded to abdominal distention in NICU, so his measured weight could not reflect the level of IGF-1 in serum accurately. Because of insufficient nutrition, his actual weight gain may be lower than it in normal infant of 29 weeks, and persistent parenteral nutrition may also increase risk for the disease independent of weight gain by absence of ω-3 long-chain polyunsaturated fatty acids (26). The infant with severe ROP undetected by CO-ROP was diagnosed with hydrocephalus which is one of the criteria in G-ROP, so similar explanation above could also be used. Since the retina is part of the central nervous system, hydrocephalus may also increase risk for the disease independently. Neither of the two infants missed by ROPscore received blood transfusion, which was different from other infants with severe ROP, although both of them had similar WG rate at 42 days with other infants with severe ROP. This may indicate that the influence of blood transfusion on ROP should be reduced in the population of our study. In addition, the two infants were both conceived by *in vitro* fertilization. Although infants by *in vitro* fertilization were more likely to develop ROP and treated ROP compared with infants by other assisted conception in previous studies (27), other studies have found that assisted conception did not appear to be a risk factor for ROP (28).

When screening models based on postnatal weight gain were used, the examination should be conducted continually according to current guidelines before the time point at which the weight of infants was needed for the application of models. For example, the risk of ROP was only defined when the weight of an infant was measured at 6 weeks after birth for ROPscore. In a word, G-ROP, CO-ROP, and ROPscore performed well for predicting severe ROP with high sensitivity, while performance for any ROP was in general when compared with other validation studies. As mentioned above, a possible reason was that more older infants with larger BW and WG after birth developed mild ROP in our population. It was worth mentioning that postnatal WG seemed to increase limited predictive performance for mild ROP in our validation, indicating the complex association of WG with ROP. For example, the association in older infants with mild ROP may be different from the association in younger infants with severe ROP (29).

There are also some limitations to our study. Firstly, only 23 to 25 infants with severe ROP were included in our study. Chinese screening criteria were GA < 32 weeks or BW < 2,000 g, which included many infants with larger GA and BW than in other studies, and in those infants with larger GA and BW, the proportion of severe ROP was low. Another cause of the low proportion of severe ROP may be the strictly controlling oxygen inhalation after birth in very low GA and BW infants. Secondly, larger GA and BW may also influence the performance of models. Thirdly, our study was a retrospective validation, so it was inevitable to exclude some infants without the necessary data who may be relatively “healthier”, which may affect the reliability of the results. However, complete data of WG for models were recorded in almost infants with severe ROP due to the enough hospitalization days, indicating the reliable prediction results of severe ROP. For any ROP, the sensitivity may be higher than it actually is, but models needed to get high sensitivity in those included infants who were relatively unwell, before they are applied to the whole and continuous population.

Although our validation was conducted using a population with about 500 to 1,000 infants in two medical centers, larger cohorts in multiple centers are required to verify the reliability of these models in Chinese cities.

## Conclusion

Strong performance of G-ROP, Co-ROP, and ROPscore for predicting severe ROP in the Chinese population was found, with sensitivity of 96%, 96%, and 91.3% respectively, while they had general prediction ability for any ROP, and the Alex-ROP model performed poorly in both severe ROP and any ROP. In the same population of 546 infants, CO-ROP (not hg CO-ROP) provided the best performance for severe ROP with 100% sensitivity and 39.9% specificity in four models. It indicated that the current ROP screening algorithm needs to be adjusted according to the characteristic of the local population and that an ROP prediction model based on postnatal weight gain for screening ROP in China needs to be developed.

## Data Availability

The original contributions presented in the study are included in the article/supplementary material, further inquiries can be directed to the corresponding author/s.
